# Satellite Remote Sensing Reveals Voluntary Cover-Crop Adoption and Crop-Rotation Hotspots in the Mississippi Alluvial Plain

**DOI:** 10.1371/journal.pone.0331797

**Published:** 2025-10-21

**Authors:** Zobaer Ahmed, Lawson Connor, Kris Brye, V. Steven Green, Mike Popp, Aaron Shew, Lawton L. Nalley

**Affiliations:** 1 Center for Advanced Spatial Technologies, University of Arkansas, Fayetteville, Arkansas, United States of America; 2 Environmental Dynamics Program, University of Arkansas, Fayetteville, Arkansas, United States of America; 3 Department of Agricultural Economics and Agribusiness, University of Arkansas, Fayetteville, Arkansas, United States of America; 4 Department of Crop, Soil, and Environmental Sciences, University of Arkansas, Fayetteville, Arkansas, United States of America; 5 College of Agriculture, Arkansas State University, Jonesboro, Arkansas, United States of America; 6 University of Arkansas System Division of Agriculture, Little Rock, Arkansas, United States of America; 7 Acres (Acres.com), Fayetteville, Arkansas, United States of America; UF: University of Florida, UNITED STATES OF AMERICA

## Abstract

Government-subsidized programs like the Environmental Quality Incentives Program and the Conservation Stewardship Program provide financial motivation for adopting cover crops. Nevertheless, many producers have internalized the holistic benefits of cover crops and voluntarily adopted them as a sustainable soil management practice. Yet, little is known about how cover crop adoption propagates beyond the first order link between financial incentives and total adopted cover crop acres. This study examined voluntary adoption of winter cover crops as well as which crop rotations had the highest cover crop use in the Mississippi Alluvial Plain (MAP) region. Cover crop locations were identified using remote sensing technologies, ground-truthed government data sources, and the United States Department of Agriculture’s Cropland Data Layer. Results in this study revealed a 5.3% increase in total voluntary cover crop adoption in the study region from 2013 to 2019. The analysis also revealed four predominant cash crop rotations associated with the use of cover crops, with a soybean/soybean rotation having the greatest association with increased wintertime cover crop acreage. Results provided valuable insights for policymakers and stakeholders to promote sustainable agricultural practices, to foster further adoption of cover crops, and optimize cover crop integration into cropping systems in the MAP and other regions.

## 1. Introduction

Winter cover crops provide benefits such as maintaining soil health, mitigating soil erosion, and improving nutrient retention in agricultural fields between primary cropping seasons [1–3]. Similarly, in the Mississippi Alluvial Plain (MAP), cover crops offer long-term benefits to producers by enhancing soil structure, increasing soil organic matter, and mitigating nutrient losses from leaching and/or runoff [4,5]. The MAP region holds great national importance due to its extensive agricultural productivity and ecological value. The MAP region encompasses parts of several states in the southern United States, including Arkansas, Mississippi, Louisiana, Missouri, and Tennessee. The region’s fertile soil and favorable climate make it a productive agricultural area known for its production of rice (*Oryza sativa* L), soybean (*Glycine max* L), cotton (*Gossypium hirsutum* L), and corn (*Zea mays* L). In other productive agricultural regions of the United States, such as the Corn belt, the adoption of wintertime cover crops experienced a significant increase of 2.3 million acres between 2006 and 2018 and occurred mainly in corn-soybean crop rotation [6]. The growth can be partially attributed to the financial incentives provided by the United States Department of Agriculture’s (USDA) Natural Resources Conservation Service (NRCS). Programs such as the Environmental Quality Incentives Program (EQIP) and Conservation Stewardship Program (CSP) have stimulated cover crop adoption as well. These federally funded programs are considered responsible for one-third of the 50% increase in the reported cover crop area in the United States between 2012 and 2017 [7]. Despite the substantial federal government expansion in funding for incentives to encourage producers to adopt cover crops since 2012 [7], growth in subsidized vs unsubsidized adoption of cover crops remains unknown due to challenges in obtaining ground-truthed, spatial data, and a reliable method for identifying voluntary adoption.

In the past decade, particularly after 2010, the utilization of remote sensing technologies have played a crucial role in effectively identifying the location and extent of wintertime cover crops across the United States [8–10]. One of the current study’s goals was to identify government-subsidized cover crops and estimate the areas of voluntary cover crops in the MAP region. The study aimed to leverage identified wintertime cover crop location data, combined with government-subsidized cover crop acreage data from EQIP and CSP programs and USDA’s Cropland Data Layer (CDL), to estimate voluntary adoption of wintertime cover crops. Further, we were able to determine what cropping systems were most common with the use of cover crops, whether voluntary or not. With a better understanding of the crop rotations where cover crop use is most common, future government funding for cover crops can be targeted more efficiently.

Cash crops planted by producers before and after planting wintertime cover crops are important to policy research and agricultural planning as the decision-making process at the farm level [11], in many parts of the MAP region, impacts soil health, pest control, and weed seedbank [12]. Further, cover crop planting is affected by the difference in timing before and after specific cash crops are harvested or planted [7]. Both late and early harvesting of cash crops may increase the duration of the cover crop season, with direct impacts on cover crop benefits and thereby cover crop adoption.

Throughout this analysis, we use the terms crop rotations and crop sequences interchangeably. However, the difference between a crop sequence and a crop rotation is important, as a crop rotation describes a patterned sequence of two or more cash crops, intended to persist for multiple years. Our study identifies a two-year sequence of cash crops and identifies whether a cover crop was planted in the winter period separating the two summer growing seasons, but does not identify whether that sequence persists beyond the identified two-year pattern.

With these concepts in mind, merely estimating the overall extent of cover crop acreage is insufficient because it gives no insight into how cover crops fit into cropping rotations, making it difficult to enhance their adoption on a broader scale. This study addresses this gap by investigating the subsidized vs unsubsidized (voluntary) adoption of cover crops in the MAP and examining producers’ crop choices before and after planting cover crops. Additionally, this research explores the potential environmental benefits of total model-predicted (government-funded vs. voluntary) cover crop adoption regarding estimated, potential soil organic carbon (SOC) sequestration, which plays a crucial role in enhancing soil health, mitigating climate change, and improving the overall sustainability of agricultural systems [13]. To our knowledge, no prior research has been undertaken to specifically identify how cover crops fit into cropping rotations using remote sensing techniques in the MAP region. This region is ideally suited for this type of analysis, as its crop diversity is greater compared to other agricultural regions in the Midwest where a corn/soybean rotation is common. This study’s findings thus provide valuable information to policymakers and agricultural stakeholders to optimize cover crop adoption and improve agricultural practices in the MAP region and beyond. We deem it necessary to study cover crop adoption over time to identify factors and crop rotations/sequences that could encourage farmers to adopt cover crops independent of government funding to uncover ‘additionality’ and ‘spillover effects’ of government cost-share funding discussed further below [14].

## 2. Materials and methods

### 2.1. Study area

**Fig 1:**
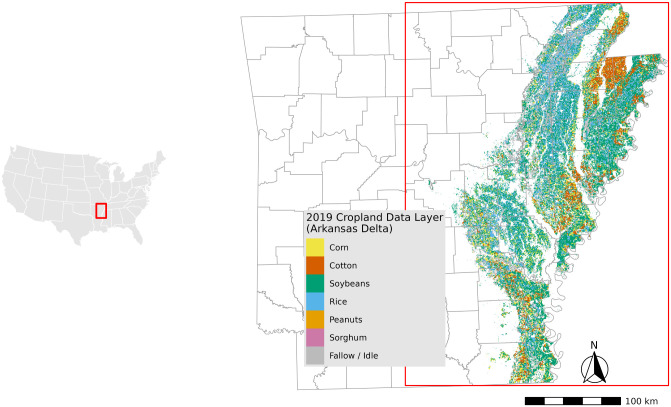
Study area: Arkansas counties in the Mississippi Alluvial Plain and re-classified 2013 CDL land-cover raster. *Source: author-generated in R Statistical Software (public-domain data); CC BY 4.0.*

The focus region for this research is the Arkansas portion of the MAP region, which includes the entirety or parts of 27 counties (Fig 1). As shown in the 2013 CDL for the region, a large variety of cash crops are grown, making the area ideally suited for distinguishing a host of land management choices.

### 2.2.Data

The data utilized in this study were derived from various sources, including remote sensing data, government-subsidized cover crop data, model-predicted areas with and without cover crop adoption, and USDA-CDL data layers. To identify the voluntary adoption of cover crops at the county level, total model-predicted wintertime cover crop county-level acreage data in conjunction with total government-subsidized cover crop acreage data from NRCS from 2013 to 2019 were used. The process for mapping the voluntary adoption of cover crops is detailed in above. Reclassified CDL data were combined with the model-predicted areas with and without cover crops to further identify crop sequences. The CDL product provides an annual, georeferenced land cover map for the continental United States, encompassing 133 different crops and non-crop raster data. The CDL data for multiple years (2013–2020) were sourced from the Google Earth Engine (GEE) data catalog. To streamline the analysis, a GEE script was used to reclassify the crop types from the CDL into seven major classes (Fig 1 illustrates an example of the reclassified CDL).

For this study, five major CDL crop categories: corn, cotton, rice, sorghum (*Sorghum bicolor* L), and soybean, were selected, as these are the predominant cash crops in the MAP region. Additionally, four double-crop pixels (i.e., double-crop winter wheat/soybean, double-crop winter wheat/corn, double-crop winter wheat/sorghum, and double-crop winter wheat/cotton) were combined into a single, double-crop category, with all remaining pixels falling into the “Minor Crops” category. Fallow/Idle land designations become part of the cropping sequence.

Finally, despite being a significant crop in the MAP study area, rice production does not typically integrate cover crops. Rice production primarily occurs on poorly drained soils, leading to saturated conditions during winter, which limits cover crop survival due to excessively wet or flooded soils. Additionally, some rice fields are intentionally flooded during winter to create a habitat for ducks, further limiting the feasibility of establishing cover crops. Furthermore, the initial analysis revealed limited acreage for rice, sorghum, and double-crop systems within cover-cropped fields. Consequently, these categories were combined under the “Other Crops” and includes “Minor Crops” as well.

### 2.3. Identification of areas with and without cover crop adoption

Agricultural crop mapping and acreage estimation are challenging given the diverse farming systems, varying field sizes/boundaries, crop heterogeneity, and heterogeneity in objectives across land management systems [15]. Despite these challenges, such mapping remains a vital prerequisite to identifying agricultural farms, their respective crops, and their spatial distribution [16]. The USDA-National Agricultural Statistics Service (NASS) combines remote sensing and field-based data to map and estimate crop acreage. They generate a range of public products, such as the CDL. However, the CDL database has limited information concerning winter cover crops, particularly their spatial and temporal distribution.

To address the data gaps and generate new information for the MAP study region, Landsat 8 satellite images were combined with yearly USDA-NRCS cover crop location data to serve as training data for a random forest machine learning model implemented in GEE to identify and map areas with and without cover crops, along with their corresponding acreage, over seven years following Ahmed et al.’s approach [17]. Specifically, the USDA-NRCS government cover crop dataset, along with the NASA Landsat 8 Operational Land Imager (OLI) Top of Atmosphere (TOA) 30-m spatial and 16-day temporal resolution remote sensing, and CDL data, allows using a pixel-based method to identify cover crops and consists of three steps. Initially, the ground-cover months (November to March) for the cover crop were identified. Next, cloud cover was eliminated from the Landsat satellite data. Finally, a machine learning algorithm was used to identify the cover crop, employing multiple spectral reflectance bands and indices. For that last step, the USDA-NRCS cover crop ground-truthed data were split into training (70%) and validation (30%) sets for the final classification using the GEE platform, where a nonparametric random forest (RF) machine learning classifier categorized the final image composites into areas with and without cover crops [8,18,19].

A limitation encountered while using the USDA-NRCS database was the occasional lack of GPS accuracy for specific cover crop locations, which occurs when producers record their GPS location from their home or farm shop rather than the actual site of the cover crop planting on their farm. A rule or threshold-based approach, similar to Ghazaryan et al. [20], was used for filtering cover crop point data to overcome the problem and ensure quality training data for machine learning models. Locations within cover crop fields were filtered and pinpointed by applying a normalized difference vegetation index (NDVI) threshold value greater than 0.3, aligning with prior studies’ methodologies [8–10]. Using the GEE platform, pixels with an NDVI value exceeding 0.3 were classified as cover crops. For the analysis, it was assumed that winter wheat was a cover crop, not for grain, within the MAP study area since specific cover crop information was unavailable from the USDA-NRCS dataset. To estimate how this assumption of winter wheat as a cover crop may affect the results (quantifying total cover crop area), the USDA-NASS data on winter wheat harvested for grain in Arkansas were used as a proxy. This proxy allowed the calculation of the percentage of winter wheat for grain by dividing the USDA-NASS winter wheat area harvested yearly by the area of total cropland. The findings indicated that only a small yearly percentage (ranging from 0.8% to 9.7%) of the winter wheat for grain may have been identified in the model as a cover crop.

In contrast, pixels falling below an NDVI value of 0.3 were categorized as “none” pixels. The “none” cover crop category encompasses bare soil areas, farmsteads, shallow and sparse vegetation, winter weeds, and some non-green crop residue pixels. By leveraging the CDL cultivated data layer to exclude non-cultivated fields, a significant portion of the pixels without cover crops were ensured to represent bare soil areas or fallow land, thereby enhancing the accuracy of the classification process. Finally, the classified map data were exported and processed using ArcGIS and RStudio for visualization and subsequent analysis. To adhere to USDA-NRCS data-sharing agreements and to ensure data privacy, classified acreages to identify crop rotation and cover crops were aggregated at the county level for subsequent mapping activities.

### 2.4. Voluntary cover crop adoption

While several programs like CSP and EQIP subsidize producers to grow cover crops and to participate in conservation efforts, not all producers who adopt cover crops receive government support for cover crop adoption [21]. The total model-predicted wintertime cover crop area and the USDA-NRCS total county-level acreage data for government-subsidized cover crops from 2013 to 2019 were used to identify the voluntary adoption of cover crops at the county level. Next, the difference was calculated between the total model-predicted cover crop acreage and the acreage of cover crop fields that received government subsidies, which allowed the isolation and the identification of voluntary cover crop acres in each county for each year of the study period to uncover patterns and trends.

### 2.5. Analysis of crop sequences

Different crop sequences for the Arkansas portion of the MAP were identified using multi-temporal satellite imagery capabilities. The large and exhaustive CDL dataset contains crop-specific information on 133 different crops and non-crop raster data. Using these data would have resulted in 35,378 unique combinations of three crop (summer crop → winter crop → summer crop) sequences to analyze. The study focused on the reclassified CDL data to streamline the analysis and account for limited acreage in many crop categories. The reclassified CDL dataset was chosen to facilitate a more manageable analysis of cropping patterns, allowing the efficient examination and interpretation of the data in subsequent steps. Specifically, the study focused on three cropping patterns revolving around cash crops and their sequential planting following cover crops and areas without cover crops. These patterns included (1) finding which cash crops were planted prior to the planting of cover crops, (2) identifying which cash crops followed cover crops, and (3) mapping all potential cash crop-cover crop-cash crop sequences by area. From these patterns, the crops and sequence combinations most often associated with wintertime cover crops were determined.

### 2.5.1. Cropping pattern (CDL Cash Crops → Cover Crops)

**Fig 2:**
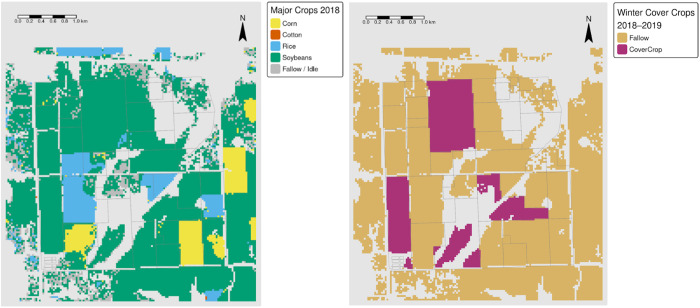
Example of CDL crops planted in areas prior to cover crops and without cover crops within the same year. CDL layer with multiple colors representing different reclassified crop types (left image) and classification of binary cover crop areas (right image). The light purple regions represent model-predicted areas with cover crops, the beige represents areas without cover crops, while the gray represents masked-out (non-cultivated) areas (right image). *Source: author-generated in R Statistical Software (public-domain data); CC BY 4.0*.

An ArcGIS combine tool was used to identify crop patterns (in this case which cash crops were planted before planting cover crops), with inputs to the ArcGIS tool consisting of reclassified CDL imagery of a specific year, succeeded by classified pixels with and without cover crops from that same year (Fig 2). This reclassification approach resulted in the generation of 14 unique crop sequence patterns per year from 2013 to 2019.

### 2.5.2. Cropping pattern (Cover Crops → CDL Cash Crops)

**Fig 3:**
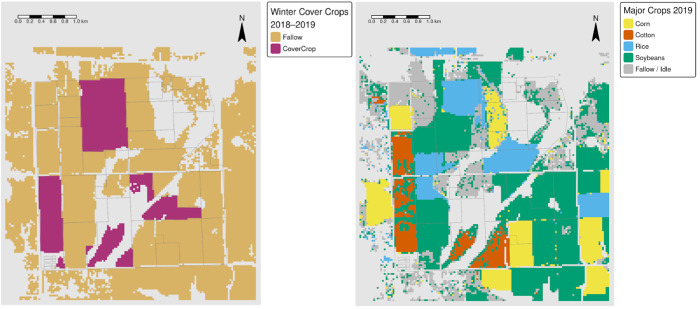
Example of CDL crops planted after a preceding cover crop and no cover crop in the following production year. CDL layer with multiple colors representing different reclassified crop types (right image) and classification of binary cover crop areas (left image). The light purple regions represent model-predicted areas with cover crops, the beige represents areas without cover crops, and the gray represents masked-out (non-cultivated) areas (left image). *Source: author-generated in R Statistical Software (public-domain data); CC BY 4.0*.

The same combination tool was applied to detect which cash crops followed cover crops. The initial input consisted of imagery with and without cover crops in a specific year, followed by reclassified CDL imagery of the following year (Fig 3). This methodology allowed for a determination of which cash crops were grown after cover crop termination, generating another 14 unique crop sequence patterns per year from 2013 to 2020.

#### 2.5.3. Crop sequences including cover crops (CDL Cash Crops → Cover Crops → CDL Cash Crops)

**Fig 4:**
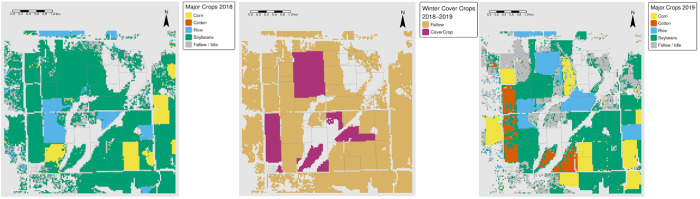
Example of cropping patterns depicting the sequence of CDL crops planted before pixels with cover crops and without cover crops within the same production year and CDL crops planted after pixels with and without cover crops, respectively, in the following year. CDL layer with multiple colors representing different reclassified crop types in the previous year (left image), binary classification of cover crops in the same year (middle image), and CDL layer with multiple colors representing different reclassified crop types in the following production year (right image). The light purple regions represent model-predicted areas with cover crops, the beige represents areas without cover crops, and the gray represents masked-out (non-cultivated) areas (middle image). *Source: author-generated in R Statistical Software (public-domain data); CC BY 4.0*.

The final cropping pattern encompassed an entire crop rotation cycle. The analysis began with the CDL cash crops of 2013, used the pixels with and without cover crops within the same year to evaluate which cash crop fields were planted to cover crops, and concluded with the CDL layer of 2014. The same procedure was followed for the other study years (Fig 4), which allowed the determination of which crop sequences were most frequently associated with the planting of cover crops. As a result, 98 distinct cropping patterns were generated per year from 2013 to 2020

### 2.6. Soil organic carbon (SOC) sequestration estimation

The methodology used to estimate potential SOC sequestration through planting cover crops incorporates several factors. Those included total cover crop acreage, biomass allocation above- or belowground given tillage practices, residue cover thresholds, tillage method used, and C concentration in the biomass. This estimation considered the total cover crop acreage across several years and delineated between government-funded and voluntarily adopted cover crop acreage.

Previous research has identified a variety of SOC sequestration rates associated with the adoption of cover crops, with these rates applicable to different soil depth intervals. Poeplau and Don [22] linked the use of cover crops to enhanced SOC sequestration and estimated a mean rate of 0.14 ± 0.04 tons/acre/year for the soil layer from 0 to 11.8 in. In contrast, Ruis & Blanco-Canqui [23] proposed a greater rate of 0.22 tons/acre/year for the same soil layer. Jian et al. [24] suggested a rate of 0.24 tons/acre/year, which applied to varying soil layers in the 0- to 11.8-in. range. It should be noted that these SOC sequestration rates primarily originated from global meta-analyses. However, it is important to recognize that SOC sequestration can vary significantly based on factors such as soil texture, agricultural management practices, elevation, climate, and location [25–27].

Blanco-Canqui [28] discussed the effects of cover crops on SOC based on a review of studies conducted in the US. Results demonstrated that cover crops accumulated SOC between 0.09 to 0.41 tons/acre/year, with an average of 0.25 tons/acre/year in the 22 instances where cover crops increased SOC. On a broader set of 77 comparisons conducted for the upper 30-in soil layer, cover crops accumulated SOC between 0 and 0.41 tons/acre/year, with an average of 0.21 tons/acre/year. Causarano et al. [29] extensively analyzed 20 studies focusing on cotton production systems in the southeastern US. The review indicated that adopting no-tillage practices compared to conventional tillage led to an average of 0.21 ± 0.25 tons/acre/year increase in SOC. Moreover, integrating high-residue-producing crops, such as corn and small grains, into diverse crop rotations further augmented SOC sequestration. Specifically, the combination of no-tillage practices with cover crops resulted in an average SOC sequestration rate of 0.30 ± 0.28 tons/acre/year, while using no-tillage practices alone yielded 0.15 ± 0.21 tons/acre/year. These results of carbon sequestration rates emphasize the significant role that cover crops and tillage practices may play in SOC sequestration initiatives within cropping systems.

While the above research provides a range of potential SOC sequestration, an alternative approach is to estimate the humification of C from stores in live vegetation. Consequently, aboveground (AG) and belowground (BG) biomasses were estimated without specific cover crop species information. The AG biomass was assumed to be 2/3 of the total biomass (B), and the BG biomass was estimated to be 1/3 of B. Using these assumptions, a conservative estimate of 3500 lbs/acre for AG and 1747 lbs/acre for BG was used for subsequent analysis [30,31].

The total AG and BG biomass in tons/acre (T_AG and T_BG, respectively) were then calculated using the respective yearly cover crop acreage (CCA) and the conversion factor from lbs to tons [Equations 1 and 2]. The combined total of AG and BG biomass (T_AG&BG) was then calculated [Equation 3].


T_AG = (AG * CCA)/2000 (1)

T_BG = (BG * CCA)/2000 (2)

T_AG&BG = T_AG + T_BG (3)

The assumption was made that 70% of the total aboveground biomass (T_AG) would be mixed into the soil (I_AG), leaving the remaining 30% as surface residue (P) to satisfy the minimum threshold to qualify for conservation tillage (CT) per USDA-NRCS guidelines [32] [Equation 4]. It is important to note that the estimated annual SOC sequestration rate might be greater than the actual rate because CT practices can also include no-tillage (NT), where 100% of the crop residue remains on the surface. However, estimating the annual SOC rate from a range of surface residue coverage was beyond the scope of the study.


I_AG = T_AG * (1−P) (4)

The total biomass in the soil (T_BS) was computed by summing I_AG and T_BG [Equation 5]. The total C input to the soil (C_S) from the cover crop biomass was then calculated by assuming a C concentration (Conc) of 50% [33] [Equation 6].


T_BS = T_BG + I_AG (5)

C_S = T_BS * Conc (6)

The biomass C requires microbial processing to achieve humification, thus, a microbial efficiency (E) of 50% was assumed [34–36]. Based on this assumption, the total soil C after microbial processing (T_SCM) was calculated [Equation 7].


T_SCM = C_S * E (7)

The final step involved calculating the SOC sequestration rate (R) in tons/acre/year by dividing T_SCM by CCA [Equation 8].


R = T_SCM/ CCA (8)

This approach, though mindful of uncertainties, aims to provide a conservative estimate of the SOC sequestration capacity of cover crops. The accuracy and applicability of these estimations depend on various factors, including the quality and representativeness of the data used, specific conditions within the study area, and management practices. Thus, more research and validation are needed to refine the estimates and enhance the understanding of the actual impact of cover crops on SOC sequestration, which will allow for a more accurate depiction of SOC sequestration rates and how they may vary across different geographies, cover crop species, and management practices. Such research would contribute to optimizing sustainable agriculture practices and climate change mitigation strategies, further underlining the value of cover crops in achieving environmental sustainability, farm profitability, and soil health improvement.

## 3. Results

### 3.1. Voluntary adoption of cover crops

**Fig 5:**
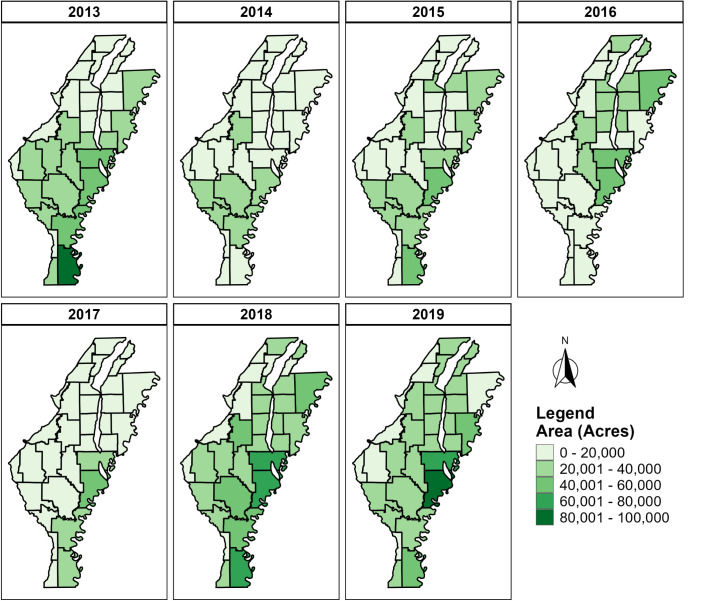
Estimated voluntary cover crop adoption acres by county across the Alluvial aquifer covering all or parts of Arkansas, Ashley, Chicot, Clay, Craighead, Crittenden, Cross, Desha, Drew, Greene, Independence, Jackson, Lawrence, Lee, Lincoln, Lonoke, Mississippi, Monroe, Phillips, Poinsett, Prairie, Pulaski, Randolph, St. Francis, White, and Woodruff counties. Source: author-generated in R Statistical Software (public-domain data); CC BY 4.0.

Both government-subsidized and voluntary cover crop adoption increased in the study area over time. According to NRCS-provided data, the government-funded total cover crop acres were lowest in 2013 (9,617 acres) and remained low until 2017 (24,386 acres) but dramatically increased in 2018 (94,464 acres) and 2019 (201,361 acres) in the MAP region. The total voluntary adoption of cover crop acreage in 2013 was 667,655 acres and increased 5% (35,608 acres) by 2019 (Fig 5). The total cover crop acres that received government cost-sharing across all counties and years amounted to 402,839 acre-years, whereas voluntary adoption totaled 4,486,197 acres. A positive correlation was found between government subsidized and voluntary adoption acres. (Pearson’s R = 0.46, P = 0.015).

Spatial analysis revealed that counties in the eastern part of the MAP region, especially those near the Mississippi River, exhibited higher adoption of cover crops compared to western counties. Cover crop adoption rates varied substantially across the region, with spatial clusters evident in areas characterized by specific cropping systems and rotations.

### 3.2. Trends in cash crops preceding and following cover crops

**Fig 6:**
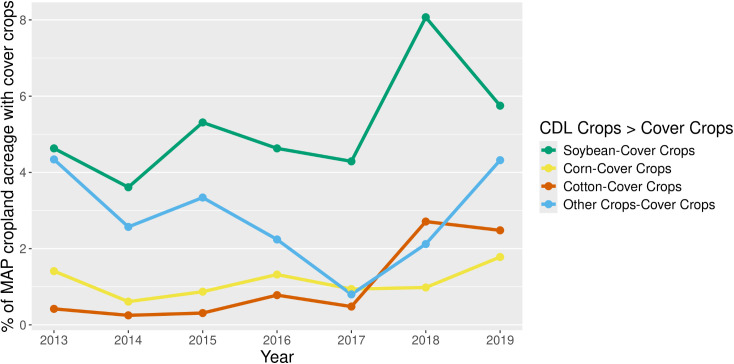
Adoption of cover crops on major summer cash crop fields. Note: This figure illustrates the percentage of acreage with cover crop over time relative to the total MAP cropland area. The calculation considers the specific cash crop planted before the cover crops in the same fields.

Analyses of the data revealed trends in the planting of winter cover crops after specific cash crops (Fig 6). Results indicated the adoption of cover crops was highest when grown following soybeans. Over the study period, the percentage of soybean acreage planted with cover crops relative to total cropland use in a particular year also rose, reaching a peak of 8% in 2018 before declining to 6% in 2019.

Like soybeans, cover crop use on corn fields also increased throughout the study period, but at a lesser adoption rate (only 1–2% of total cropland). The use of cover crops following cotton also exhibited an upward trend. In 2013, cover crops in cotton production accounted for < 0.5% of the total cropland area, but by 2018, this figure had risen to nearly 3% (Fig 6). The remaining crops from the dataset, including rice, sorghum, double crops, and other minor crops, were combined into an “Other Crops” category. The order of importance in terms of total cropland use closely follows soybean being the leading crop in average planted acreage over the period (3.2 million acres), followed by rice at 1.3, corn at 0.7, and cotton at 0.4 million acres [37].

In terms adoption rate of cover crop within a particular crop, corn and cotton had the largest percentage with a peak of 17% and 39%, respectively (S1 Fig.). While the importance of total cover crops in soybeans was notable, the maximum percentage of soybean area planted to cover crops after soybean was 14%.

**Fig 7:**
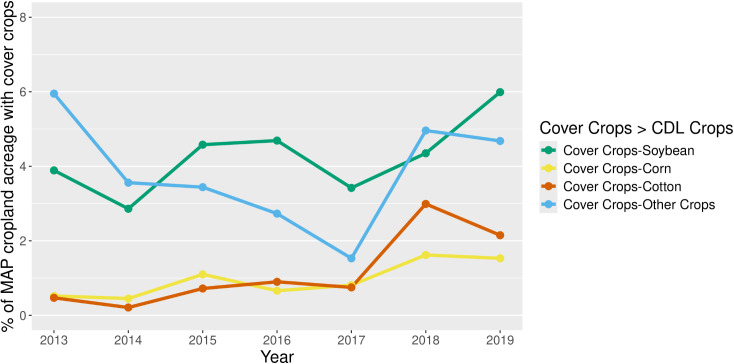
Cash crop succession following winter cover crop fields. Note: This figure showcases the percentage of acreage with cover crop over time, meaning cash crops planted exclusively to winter cover crop fields in relation to the MAP total cropland area. The analysis considers the specific cash crops planted after the termination of winter cover crops in the same fields.

Fig 7 presents the type of cash crop that followed after the cover crop. In 2013, soybean fields that succeeded winter cover crops accounted for nearly 4% of the total cropland acreage. The proportion of soybean planting in cover cropped areas fluctuated, reaching a peak of 6% in 2019. Similarly, corn and cotton fields that followed winter cover crops exhibited varying but low planting rates. The “Other Crops” planting rate following winter cover crops was 6% in 2013, which decreased to slightly less than 5% by 2019.

With respect to specific crop’s harvested acres following cover crops, corn and cotton showed the largest percentages, reaching maximum values of 18% and 34%, respectively (S2 Fig). Soybean showed an upward trend with as much as 13% of soybean harvested acreage following a cover crop.

### 3.3 Analysis of cropping sequences (CDL Cash Crops → Cover Crops → CDL Cash Crops)

**Fig 8:**
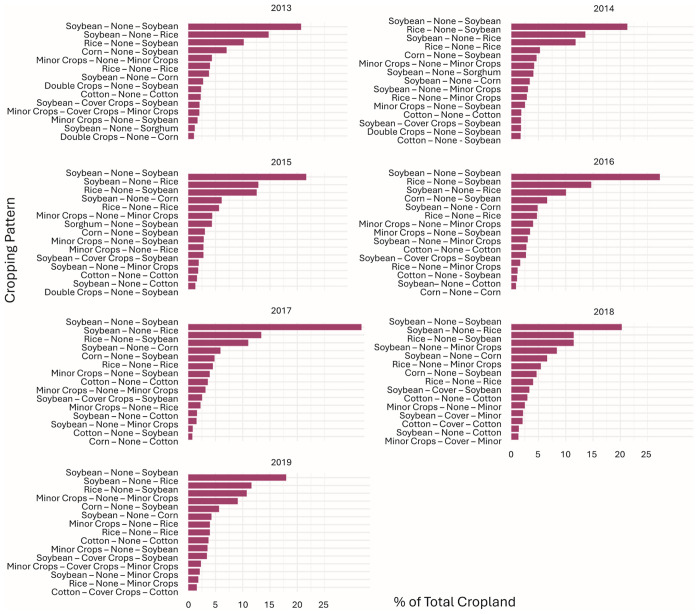
Cropping patterns for 2013 and 2019. Cash crops planted before and after winter cover crops and areas without cover crops (the top 15 cropping pattern combinations). As a result of significant rice acreage with no preceding cover crop in the region, rice acres were separated from minor crops for this analysis. Note: S1 Fig suggests that corn and cotton had the highest acreage before the introduction of a cover crop. However, Fig 8 reveals that corn is not among the top 15 cropping pattern combinations when cover crops are planted. This observation likely indicates that corn is part of a more diverse rotation pattern when cover crops are integrated.

In 2013, the predominant cropping sequence involved soybean-soybean production without cover crops, which accounted for 21% of the total cropland area (Fig 8). Rice emerged as the second most prominent cash crop after non-cover crops, accounting for 15% of the total cropland area (Table 1). Other combinations of cash crops and non-cover crops, such as rice followed by soybean and corn followed by soybean, were also predominant cropping sequences, representing 10 and 7% of the total cropland area, respectively (Table 1).

**Table 1:** Percentage of total MAP area consisting of crop rotations with and without cover crops (CC) from 2013 to 2019 sorted by first cash crop.

Cropping Pattern	2013	2014	2015	2016	2017	2018	2019
Corn – None – Corn	0	0	0	0.88	0	0	0
Corn – None – Cotton	0	0	0	0	0.75	0	0
Corn – None – Soybean	7.06	4.66	3.05	6.57	4.81	4.65	5.65
Cotton – CC – Cotton	0	0	0	0	0	2.09	1.59
Cotton – None – Cotton	2.36	1.82	1.81	2.76	3.58	2.94	3.71
Cotton – None – Soybean	0	1.7	0	1.16	0.83	0	0
Double Crops – None – Corn	1.04	0	0	0	0	0	0
Double Crops – None – Soybean	2.73	1.77	1.31	0	0	0	0
Minor Crops – CC – Minor Crops	2.06	0	0	0	0	1.31	2.33
Minor Crops – None – Minor Crops	4.33	4.19	4.39	4	3.14	2.49	9.1
Minor Crops – None – Rice	0	0	2.79	0	2.25	0	3.97
Minor Crops – None – Soybean	2.03	2.5	2.85	3.44	3.92	0	3.52
Rice – None – Minor Crops	0	2.84	0	1.64	0	5.41	1.86
Rice – None – Rice	3.99	5.25	5.65	4.7	4.52	4	3.95
Rice – None – Soybean	10.17	13.59	12.55	14.65	11	11.46	10.71
Sorghum – None – Soybean	0	0	4.33	0	0	0	0
Soybean – CC – Minor Crops	0	0	0	0	0	2.14	0
Soybean – CC – Soybean	2.3	1.77	2.77	2.71	2.52	3.32	3.43
Soybean – None – Corn	3.8	3.39	6.13	4.82	5.89	6.55	4.26
Soybean – None – Cotton	0	0	1.6	1.07	1.62	1.38	0
Soybean – None – Minor Crops	1.69	3.06	1.92	3.05	1.51	8.35	2.1
Soybean – None – Rice	14.73	11.79	12.86	10.01	13.37	11.46	11.59
Soybean – None – Sorghum	1.21	4.07	0	0	0	0	0
Soybean – None – Soybean	20.72	21.26	21.63	27.24	31.77	20.24	17.95

Note: Please see Appendix Table S1 for the statistical significance of interannual trends in these cropping patterns.

By 2019, there was a shift in crop rotations compared to 2013 (Fig 8). Soybean, though still the most common crop grown after non-cover crops, dropped to 18% of the cropland area, down from 21% in 2013. The emergence of cover crops between soybean production, denoted as “Soybean - Cover Crops - Soybean,” accounted for 3% of the total cropland area in 2019. Additionally, combining cover crops with other cash crops, such as “Minor Crops - Cover Crops - Minor Crops,” represented 2% of the total cropland area.

A total of four unique cropping sequences utilizing cover crops were identified across all years: Cotton – Cover Crop – Cotton, Minor Crops – Cover Crop – Minor Crops, Soybean – Cover Crops – Minor Crops, and Soybean – Cover Crops – Soybean, with the latter being the earliest, most consistent and largest (Fig 8)

### 3.4 Soil organic carbon (SOC) sequestration potential from cover cropped area

Soil organic carbon sequestration estimates associated with cover crop adoption increased over time (Table 2). In 2013, total SOC sequestration from both government-subsidized and voluntary cover crop acres (677,272 acres) was approximately 355,346 tons of carbon. By 2019, this amount increased 34%, reaching 474,631 tons. Government-funded acres notably increased from contributing 5,046 tons of carbon in 2013 to 105,648 tons in 2019. In contrast, voluntary SOC sequestration increased more modestly, rising approximately 5% from 350,300 tons in 2013 to 368,982 tons in 2019. The estimated SOC sequestration rate applied was a conservative 0.52 tons C acre ⁻ ¹ year ⁻ ¹ (1.16 Mg ha ⁻ ¹ year ⁻ ¹; see Equation 1), with a more conservative alternate estimate from Poeplau and Don [22] provided at 0.14 tons C acre ⁻ ¹ year ⁻ ¹ (0.32 Mg ha ⁻ ¹ year ⁻ ¹).

**Table 2:** Estimated Soil Organic Carbon (SOC) sequestration from cover crop adoption (2013-2019).

Year	Government CCA (acres)	Government SOC (tons)	Voluntary CCA (acres)	Voluntary SOC (tons)	Total CCA (acres)	Total SOC (tons)
2013	9,617	5,046	667,655	350,300	677,272	355,346
2014	18,758	9,842	421,908	221,363	440,666	231,205
2015	30,364	15,931	584,722	306,787	615,086	322,718
2016	23,889	12,534	537,644	282,087	561,533	294,621
2017	24,386	12,795	382,446	200,659	406,832	213,453
2018	94,465	49,563	785,720	412,245	880,185	461,808
2019	201,361	105,648	703,263	368,982	904,624	474,631

Note: Equations 1–8 were utilized to estimate the SOC sequestration values.

## 4. Discussion

The fluctuations in cover crop acreage from year to year, observed for both government‑subsidized and voluntary adoption, could be attributed to a combination of external factors. Notably, government‑subsidized cover crop acreage in the study area saw a surge post‑2013 and 2018, which may be related to policy changes. The Farm Bills of 2014 and 2018 likely played a role in this uptick as they provided farmers with financial incentives and technical assistance to adopt conservation practices, including cover cropping [38,39]. Additionally, it is worth noting that even farmers who are big supporters of cover crops might not use cover crops on all their fields every year. Furthermore, due to variations in crop rotation and/or cropping sequences from year to year and variations in weather patterns, there is not always a guarantee that cover crops will be present during the winter period.

The positive correlation between government cost‑shared and voluntary adoption signifies that, in the study area, producers either voluntarily chose to adopt cover crops on their land without government assistance or subsidies, or there were cases where farmers had originally adopted cover crops with government support and chose to continue using them after the government assistance ended. This could be influenced by similar cropping rotations within these counties wherein producers would find cover crops beneficial. This finding suggests that the financial support provided through programs like EQIP and CSP may have played a role in encouraging increased adoption of cover crops, which is backed by previous literature [16,40]. Furthermore, study results suggest the presence of ‘spillover effects’ wherein the practices adopted by farmers benefiting from government programs could influence and inspire other farmers to adopt cover crops voluntarily [16,40].

The notable prevalence of cover crop adoption in counties near the Mississippi River can likely be attributed to the kinds of crops typically cultivated in the eastern MAP region and the cropping rotations used. A study that spanned several states in the upper Midwestern Mississippi River Basin reported that 34–81% of agricultural land in select counties had the potential for integrating cover crops into corn and soybean crop systems [4]. Other potential contributing factors include farmer conservation attitudes, regional climate conditions, local geographical features, agricultural market dynamics, incentive programs, and the influences of social networks. A wealth of research studies focusing on the MAP region have highlighted the advantages of integrating cover crops into these farming systems, notably enhancing soil health, mitigating soil erosion, and enhancing weed control [41–43].

The government’s support for environmentally conscious production practices and subsidies for cover crops may have motivated farmers to embrace these practices voluntarily [44]. Voluntary adopters may have recognized the long‑term benefits of cover crops regarding soil health, environmental conservation, and lowering farming expenses [45,46]. However, the complex and time‑consuming application procedures and detailed record‑keeping associated with government assistance and incentive programs may have discouraged many farmers from seeking financial support [47]. This trend highlights a growing awareness among producers regarding the potential benefits and value associated with cover crops. Producers could be motivated to voluntarily increase adoption, even without financial incentives. Moreover, producers may have come to realize that these practices, such as using cover crops, could contribute to carbon offset initiatives and potentially generate carbon credits, further adding to their appeal [40,48–50].

These findings indicate growth in the use of cover crops among soybean producers. Soybean cropping systems benefit greatly from cover crop use to keep soil in place because soybean is generally considered a low‑biomass‑producing crop that does not return enough residue after harvest to provide adequate soil erosion protection. Additionally, given soybeans are typically planted as late as June in the MAP region, while other cash crops such as corn may be planted as early as February, termination guidelines for cover crops in the MAP region may allow for greater biomass accumulation of the cover crop when planted with soybeans compared to other cash crops.

Although the inclusion rate of cover crops on corn fields remained lower than on soybean fields, results suggested an increasing acceptance of cover crops among corn producers. The increased planting of cover crops on corn acres could be attributed to farmers’ acknowledgment of the production advantages they provide specifically for corn production. In contrast to soybean, corn is a large biomass‑producing crop, thus requiring greater soil manipulation by tillage to manage the greater amount of surface residue when no‑tillage practices are not used, thus increasing the likelihood of soil disturbance and the potential for off‑site soil transport. Consequently, cover crop use provides additional protection from potential soil erosion. In addition, as a crop that requires large fertilizer‑N additions for optimal production, using legumes in a cover crop mix can increase soil N for subsequent crop use.

Since cotton is a woody perennial, the residue left behind after harvest is typically low in quality. Cover crops diversify this residue’s quality and augment the substrate quantity, enhancing soil health and soil organic matter (SOM). This substrate can boost soil health through SOC sequestration more quickly than without cover crops. Moreover, numerous cotton producers utilize cover crops to enhance soil moisture and conserve water during the cotton growing season by using cover crop residue as ground cover.

According to survey data analyses conducted by Wallander et al., the use of winter cover crops has increased across the US [7]. Approximately 5% of corn acreage, as of 2016, had implemented cover crops, while the cover crop rate was slightly greater for soybeans at 8% as of 2018 [7]. As of 2015, the rate of cover crops in cotton fields was roughly 13% [7]. Results align with the increasing trends regarding the use of cover crops, suggesting that farmers are increasingly aware of the potential benefits of cover crop use.

The analysis unveiled varying planting rates of cash crops following winter cover crops, supported by findings from previous studies [51–54]. The observed increasing trend in the inclusion of winter cover crops within soybean, corn, and cotton cropping systems demonstrates farmers’ recognition of the potential benefits, including enhanced soil health and the promotion of sustainable agricultural practices [55–57]. Encouraging more comprehensive implementation of winter cover crops across these major cash crops can significantly contribute to the overall sustainability and resilience of agricultural systems. However, further research is necessary to investigate the factors influencing adoption rates and to evaluate the long‑term impacts of integrating cover crops into cash crop rotations [46,58].

These findings indicate a shift in cropping patterns from 2013 to 2019, reflecting the increasing use of cover crops between cash crops. This trend signifies evolving agricultural practices regionally and the recognition of the potential benefits of cover crops, including improved soil health and erosion control. The observed changes in cropping patterns highlight the dynamic nature of the agricultural landscape in the MAP region, with farmers adapting their cash crop choices based on the presence of winter cover crops over time and the cash crops’ prices. These shifts may be influenced by market demands, agronomic considerations, and policy changes. Market demands can significantly shape cropping rotations as farmers respond to changing market conditions. Agronomic considerations such as soil fertility, pest management, and crop rotation also influence crop choices and the integration of cover crops. Farmers may adjust their cropping rotations based on the need to replenish soil nutrients, control pests and weeds, or break disease cycles.

Local, regional, or national policy changes can substantially impact cropping patterns. Government programs such as EQIP and CSP, incentives, or regulations that promote the adoption of cover crops or sustainable agricultural practices can influence farmers’ decision‑making processes. Likewise, changes in agricultural policies related to subsidies, crop insurance, or conservation programs can incentivize or discourage specific cropping patterns.

Understanding shifts in cropping patterns, including the increased adoption of cover crops, is crucial for stakeholders and policymakers in developing resilient and sustainable agriculture strategies. Recognizing the factors behind these changes allows for targeted interventions, such as financial incentives or educational programs, to foster sustainable farming practices. Collaboration between stakeholders like producers, researchers, and agricultural extension services in spreading information on cover crops’ benefits can promote best practices, enhance knowledge‑sharing, and facilitate implementation. This cooperative approach can augment agricultural systems’ long‑term viability and resilience.

It is important to note that including the model‑predicted total cover crop acreage, assumed biomass allocation, residue cover thresholds, and C concentration in biomass used for calculating the SOC tons sequestered in this study was primarily intended to provide a conservative estimate for context rather than represent a definitive estimate. The sequestration potential can vary significantly due to specific crop types, management practices, climatic conditions, and numerous soil characteristics, especially soil texture. Future research should incorporate site‑specific data and consider these influential factors carefully to obtain more precise SOC sequestration estimations. By doing so, the accuracy and reliability of assessments regarding SOC sequestration in the context of cover crop adoption can be significantly enhanced.

Further research, incorporating more advanced modeling approaches and considering site‑specific factors, is needed to obtain a more accurate assessment of SOC dynamics and the true potential of cover crop adoption for soil C sequestration. Additionally, it is crucial to expand the analysis beyond SOC sequestration and consider the broader ecosystem benefits associated with cover crop adoption. Cover crops are vital in enhancing soil health, improving water quality, reducing erosion, and promoting biodiversity. Understanding the interconnections between these ecological processes and SOC sequestration will provide a more comprehensive evaluation of the overall impact of cover crop adoption on ecosystem services. However, at present, SOC sequestration is important to at least estimate due to its implication for C credits as a tradeable commodity in developing markets.

In summary, our analysis confirms that cover crops are gaining traction as a multifaceted strategy, enhancing soil health and erosion control, offering yield and nutrient benefits, and unlocking emerging carbon credit revenues through both government cost share programs and voluntary adoption. Despite county level data and remote sensing limitations, leveraging NRCS records and imagery proved effective for tracking adoption trends and evolving cash crop rotations, with soybean systems leading uptake. The conservative SOC sequestration estimates of 0.52 tons of carbon per acre per year underscore cover crops’ potential role in carbon offset initiatives, while the rapid, replicable data collection framework offers a valuable tool for policymakers and researchers. Moving forward, targeted collaboration among farmers, extension agents, and public and private partners will be essential to refine farm practices, tailor incentive programs, including emerging voluntary carbon markets, and scale cover crop systems for resilient, sustainable agriculture across the MAP region and beyond.

## Supporting Information

S1 Fig Temporal trend of the percentage of each crop in the MAP region when followed by a cover crop. (TIF)

S2 Fig Temporal trend of the percentage of each crop in the MAP region after a cover crop. (TIF)

S1 Table Statistical significance of interannual trends in cover crop (CC) adoption by crop sequence in order first cash crop. (DOCX)

S1 Data Raw Data. (XLSX)

## References

[1] Basche AD, Archontoulis SV, Kaspar TC, Jaynes DB, Parkin TB, Miguez FE. Simulating long-term impacts of cover crops and climate change on crop production and environmental outcomes in the Midwestern United States. Agriculture, Ecosystems & Environment. 2016;218:95–106. doi: 10.1016/j.agee.2015.11.011

[2] Dabney SM, Delgado JA, Reeves DW. USING WINTER COVER CROPS TO IMPROVE SOIL AND WATER QUALITY. Communications in Soil Science and Plant Analysis. 2001;32(7–8):1221–50. doi: 10.1081/css-100104110

[3] Adetunji AT, Ncube B, Mulidzi R, Lewu FB. Management impact and benefit of cover crops on soil quality: A review. Soil and Tillage Research. 2020;204:104717. doi: 10.1016/j.still.2020.104717

[4] Kladivko EJ, Kaspar TC, Jaynes DB, Malone RW, Singer J, Morin XK, et al. Cover crops in the upper midwestern United States: Potential adoption and reduction of nitrate leaching in the Mississippi River Basin. Journal of Soil and Water Conservation. 2014;69(4):279–91. doi: 10.2489/jswc.69.4.279

[5] Aryal N, Reba ML, Straitt N, Teague TG, Bouldin J, Dabney S. Impact of cover crop and season on nutrients and sediment in runoff water measured at the edge of fields in the Mississippi Delta of Arkansas. Journal of Soil and Water Conservation. 2018;73(1):24–34. doi: 10.2489/jswc.73.1.24

[6] Geosolutions AL, Dagan I, The Nature Conservancy, CTIC. Mapping conservation practices and outcomes in the corn belt - final report. 2019. https://www.ctic.org/files/FinalReport_CBPPP_CTIC-TNC_v2.pdf

[7] Wallander S, Smith D, Bowman M, Claassen R. Cover Crop Trends, Programs, and Practices in the United States. 2021. https://www.ers.usda.gov/publications/pub-details/?pubid=100550

[8] Kc K, Zhao K, Romanko M, Khanal S. Assessment of the Spatial and Temporal Patterns of Cover Crops Using Remote Sensing. Remote Sensing. 2021;13(14):2689. doi: 10.3390/rs13142689

[9] Hively WD, Duiker S, McCarty G, Prabhakara K. Remote sensing to monitor cover crop adoption in southeastern Pennsylvania. Journal of Soil and Water Conservation. 2015;70(6):340–52. doi: 10.2489/jswc.70.6.340

[10] Thieme A, Yadav S, Oddo PC, Fitz JM, McCartney S, King L, et al. Using NASA Earth observations and Google Earth Engine to map winter cover crop conservation performance in the Chesapeake Bay watershed. Remote Sensing of Environment. 2020;248:111943. doi: 10.1016/j.rse.2020.111943

[11] Dury J, Schaller N, Garcia F, Reynaud A, Bergez JE. Models to support cropping plan and crop rotation decisions. A review. Agron Sustain Dev. 2011;32(2):567–80. doi: 10.1007/s13593-011-0037-x

[12] Bergtold JS, Ramsey S, Maddy L, Williams JR. A review of economic considerations for cover crops as a conservation practice. Renew Agric Food Syst. 2017;34(1):62–76. doi: 10.1017/s1742170517000278

[13] Qin Z, Guan K, Zhou W, Peng B, Tang J, Jin Z, et al. Assessing long-term impacts of cover crops on soil organic carbon in the central US Midwestern agroecosystems. Glob Chang Biol. 2023;29(9):2572–90. doi: 10.1111/gcb.16632 36764676

[14] Mezzatesta M, Newburn DA, Woodward RT. Additionality and the Adoption of Farm Conservation Practices. Land Economics. 2013;89(4):722–42. doi: 10.3368/le.89.4.722

[15] Liu L, Xiao X, Qin Y, Wang J, Xu X, Hu Y, et al. Mapping cropping intensity in China using time series Landsat and Sentinel-2 images and Google Earth Engine. Remote Sensing of Environment. 2020;239:111624. doi: 10.1016/j.rse.2019.111624

[16] Hudait M, Patel PP. Crop-type mapping and acreage estimation in smallholding plots using Sentinel-2 images and machine learning algorithms: Some comparisons. The Egyptian Journal of Remote Sensing and Space Science. 2022;25(1):147–56. doi: 10.1016/j.ejrs.2022.01.004

[17] Ahmed Z, Nalley L, Brye K, Green VS, Popp M, Shew AM, et al. Winter-time cover crop identification: A remote sensing-based methodological framework for new and rapid data generation. International Journal of Applied Earth Observation and Geoinformation. 2023;125:103564. doi: 10.1016/j.jag.2023.103564

[18] Ok AO, Akar O, Gungor O. Evaluation of random forest method for agricultural crop classification. European Journal of Remote Sensing. 2012;45(1):421–32. doi: 10.5721/eujrs20124535

[19] Pal M. Random forest classifier for remote sensing classification. International Journal of Remote Sensing. 2005;26(1):217–22. doi: 10.1080/01431160412331269698

[20] Ghazaryan G, Dubovyk O, Löw F, Lavreniuk M, Kolotii A, Schellberg J, et al. A rule-based approach for crop identification using multi-temporal and multi-sensor phenological metrics. European Journal of Remote Sensing. 2018;51(1):511–24. doi: 10.1080/22797254.2018.1455540

[21] Dunn M, Ulrich-Schad JD, Prokopy LS, Myers RL, Watts CR, Scanlon K. Perceptions and use of cover crops among early adopters: Findings from a national survey. Journal of Soil and Water Conservation. 2016;71(1):29–40. doi: 10.2489/jswc.71.1.29

[22] Poeplau C, Don A. Carbon sequestration in agricultural soils via cultivation of cover crops – A meta-analysis. Agriculture, Ecosystems & Environment. 2015;200:33–41. doi: 10.1016/j.agee.2014.10.024

[23] Ruis SJ, Blanco‐Canqui H. Cover Crops Could Offset Crop Residue Removal Effects on Soil Carbon and Other Properties: A Review. Agronomy Journal. 2017;109(5):1785–805. doi: 10.2134/agronj2016.12.0735

[24] Jian J, Lester BJ, Du X, Reiter MS, Stewart RD. A calculator to quantify cover crop effects on soil health and productivity. Soil and Tillage Research. 2020;199:104575. doi: 10.1016/j.still.2020.104575

[25] Bai X, Huang Y, Ren W, Coyne M, Jacinthe P-A, Tao B, et al. Responses of soil carbon sequestration to climate-smart agriculture practices: A meta-analysis. Glob Chang Biol. 2019;25(8):2591–606. doi: 10.1111/gcb.14658 31002465

[26] Lessmann M, Ros GH, Young MD, de Vries W. Global variation in soil carbon sequestration potential through improved cropland management. Glob Chang Biol. 2022;28(3):1162–77. doi: 10.1111/gcb.15954 34726814 PMC9299007

[27] Herzfeld T, Heinke J, Rolinski S, Müller C. SOC sequestration potentials for agricultural management practices under climate change. Copernicus GmbH. 2021. doi: 10.5194/esd-2021-35

[28] Blanco‐Canqui H. Cover crops and carbon sequestration: Lessons from U.S. studies. Soil Science Soc of Amer J. 2022;86(3):501–19. doi: 10.1002/saj2.20378

[29] Causarano HJ, Franzluebbers AJ, Reeves DW, Shaw JN. Soil organic carbon sequestration in cotton production systems of the southeastern United States: a review. J Environ Qual. 2006;35(4):1374–83. doi: 10.2134/jeq2005.0150 16825457

[30] USDA-NRCS. Estimating cover crop biomass. 2018. https://www.nrcs.usda.gov/sites/default/files/2022-09/EstBiomassCoverCrops_Sept2018.pdf

[31] Brye KR. Long-term Effects of Aboveground Biomass Removal by Burning on Potential Nutrient Recycling. Crop Management. 2012;11(1):1–9. doi: 10.1094/cm-2012-0822-01-rs

[32] Prabhakara K, Hively WD, McCarty GW. Evaluating the relationship between biomass, percent groundcover and remote sensing indices across six winter cover crop fields in Maryland, United States. International Journal of Applied Earth Observation and Geoinformation. 2015;39:88–102. doi: 10.1016/j.jag.2015.03.002

[33] Popp M, Nalley L, Fortin C, Smith A, Brye K. Estimating Net Carbon Emissions and Agricultural Response to Potential Carbon Offset Policies. Agronomy Journal. 2011;103(4):1132–43. doi: 10.2134/agronj2010.0517

[34] Saifuddin M, Bhatnagar JM, Segrè D, Finzi AC. Microbial carbon use efficiency predicted from genome-scale metabolic models. Nat Commun. 2019;10(1):3568. doi: 10.1038/s41467-019-11488-z 31395870 PMC6687798

[35] Sinsabaugh RL, Turner BL, Talbot JM, Waring BG, Powers JS, Kuske CR, et al. Stoichiometry of microbial carbon use efficiency in soils. Ecological Monographs. 2016;86(2):172–89. doi: 10.1890/15-2110.1

[36] Keiblinger KM, Hall EK, Wanek W, Szukics U, Hämmerle I, Ellersdorfer G, et al. The effect of resource quantity and resource stoichiometry on microbial carbon-use-efficiency. FEMS Microbiol Ecol. 2010;73(3):430–40. doi: 10.1111/j.1574-6941.2010.00912.x 20550579

[37] USDA NASS. Annual Planted Acres in Arkansas. https://quickstats.nass.usda.gov/results/3AFE2C85-89C3-3870-A070-3371803BA297

[38] USDA ERS. Agricultural Act of 2014: Highlights and Implications. 2014. https://www.ers.usda.gov/agricultural-act-of-2014-highlights-and-implications/

[39] USDA ERS. Agriculture Improvement Act of 2018: Highlights and Implications. 2018. https://www.congress.gov/115/bills/hr2/BILLS-115hr2enr.pdf

[40] Plastina A, Sawadgo W. Cover crops and no-till in the I-states: non-permanence and carbon markets. Agricultural Policy Review. 2021.

[41] Jacobs AA, Evans RS, Allison JK, Garner ER, Kingery WL, McCulley RL. Cover crops and no-tillage reduce crop production costs and soil loss, compensating for lack of short-term soil quality improvement in a maize and soybean production system. Soil and Tillage Research. 2022;218:105310. doi: 10.1016/j.still.2021.105310

[42] Reba ML, Aryal N, Teague TG, Massey JH. Initial findings from agricultural water quality monitoring at the edge-of-field in Arkansas. Journal of Soil and Water Conservation. 2020;75(3):291–303. doi: 10.2489/jswc.75.3.291

[43] Adler RL, Singh G, Nelson KA, Weirich J, Motavalli PP, Miles RJ. Cover crop impact on crop production and nutrient loss in a no-till terrace topography. Journal of Soil and Water Conservation. 2020;75(2):153–65. doi: 10.2489/jswc.75.2.153

[44] Park B, Rejesus RM, Aglasan S, Che Y, Hagen SC, Salas W. Payments from agricultural conservation programs and cover crop adoption. Applied Eco Perspectives Pol. 2022;45(2):984–1007. doi: 10.1002/aepp.13248

[45] Lee S, McCann L. Adoption of Cover Crops by U.S. Soybean Producers. J Agric Appl Econ. 2019;51(04):527–44. doi: 10.1017/aae.2019.20

[46] Thompson NM, Reeling CJ, Fleckenstein MR, Prokopy LS, Armstrong SD. Examining intensity of conservation practice adoption: Evidence from cover crop use on U.S. Midwest farms. Food Policy. 2021;101:102054. doi: 10.1016/j.foodpol.2021.102054

[47] Reimer AP, Prokopy LS. Farmer participation in U.S. Farm Bill conservation programs. Environ Manage. 2014;53(2):318–32. doi: 10.1007/s00267-013-0184-8 24114348

[48] Weinberg M, Claassen R. Conservation Program Design Rewarding Farm Practices versus Environmental Performance. 2006. https://www.ers.usda.gov/webdocs/publications/42913/29515_eb5_002.pdf?v=0

[49] Winsten JR, Hunter M. Using pay-for-performance conservation to address the challenges of the next farm bill. Journal of Soil and Water Conservation. 2011;66(4). doi: 10.2489/jswc.66.4.111a

[50] Bowman M, Lynch L. Government Programs that Support Farmer Adoption of Soil Health Practices: A Focus on Maryland’s Agricultural Water Quality Cost-Share Program. CHOICES. 201934. Available: https://www.choicesmagazine.org/UserFiles/file/cmsarticle_695.pdf. Accessed 2 Jun 2023.

[51] Marcillo GS, Miguez FE. Corn yield response to winter cover crops: An updated meta-analysis. Journal of Soil and Water Conservation. 2017;72(3):226–39. doi: 10.2489/jswc.72.3.226

[52] Wauters VM, Grossman JM, Pfeiffer A, Cala R. Ecosystem Services and Cash Crop Tradeoffs of Summer Cover Crops in Northern Region Organic Vegetable Rotations. Front Sustain Food Syst. 2021;5. doi: 10.3389/fsufs.2021.635955

[53] Wyland LJ, Jackson LE, Chaney WE, Klonsky K, Koike ST, Kimple B. Winter cover crops in a vegetable cropping system: Impacts on nitrate leaching, soil water, crop yield, pests and management costs. Agriculture, Ecosystems & Environment. 1996;59(1–2):1–17. doi: 10.1016/0167-8809(96)01048-1

[54] Almoussawi A, Lenoir J, Spicher F, Dupont F, Chabrerie O, Closset-Kopp D, et al. Direct seeding associated with a mixture of winter cover crops decreases weed abundance while increasing cash-crop yields. Soil and Tillage Research. 2020;200:104622. doi: 10.1016/j.still.2020.104622

[55] Sarrantonio M, Gallandt E. The Role of Cover Crops in North American Cropping Systems. Journal of Crop Production. 2003;8(1–2):53–74. doi: 10.1300/j144v08n01_04

[56] Kuo S, Huang B, Bembenek R. Effect of winter cover crops on soil nitrogen availability, corn yield, and nitrate leaching. ScientificWorldJournal. 2001;1 Suppl 2:22–9. doi: 10.1100/tsw.2001.267 12805863 PMC6084230

[57] Blanco‐Canqui H, Shaver TM, Lindquist JL, Shapiro CA, Elmore RW, Francis CA, et al. Cover Crops and Ecosystem Services: Insights from Studies in Temperate Soils. Agronomy Journal. 2015;107(6):2449–74. doi: 10.2134/agronj15.0086

[58] Bergtold JS, Duffy PA, Hite D, Raper RL. Demographic and Management Factors Affecting the Adoption and Perceived Yield Benefit of Winter Cover Crops in the Southeast. J Agric Appl Econ. 2012;44(1):99–116. doi: 10.1017/s1074070800000195

