# Supplementary figures and images for: Satellite Remote Sensing Reveals Voluntary Cover-Crop Adoption and Crop-Rotation Hotspots in the Mississippi Alluvial Plain
Source: PLoS One. 2025 Oct 21;20(10):e0331797. doi: 10.1371/journal.pone.0331797 (PMC12539724; doi:10.1371/journal.pone.0331797)

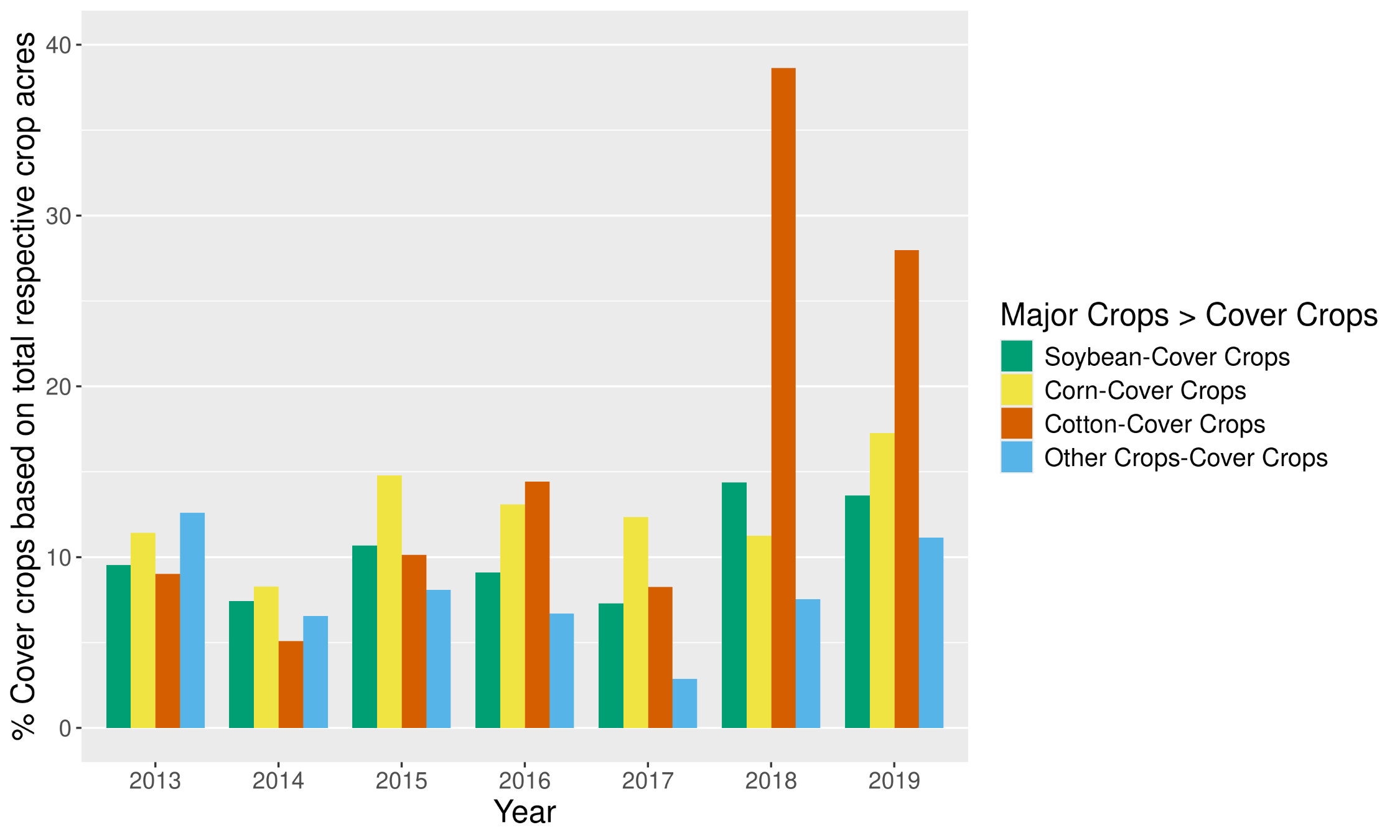

Supplement: S1 Fig — (TIF) [file pone.0331797.s001.tif]

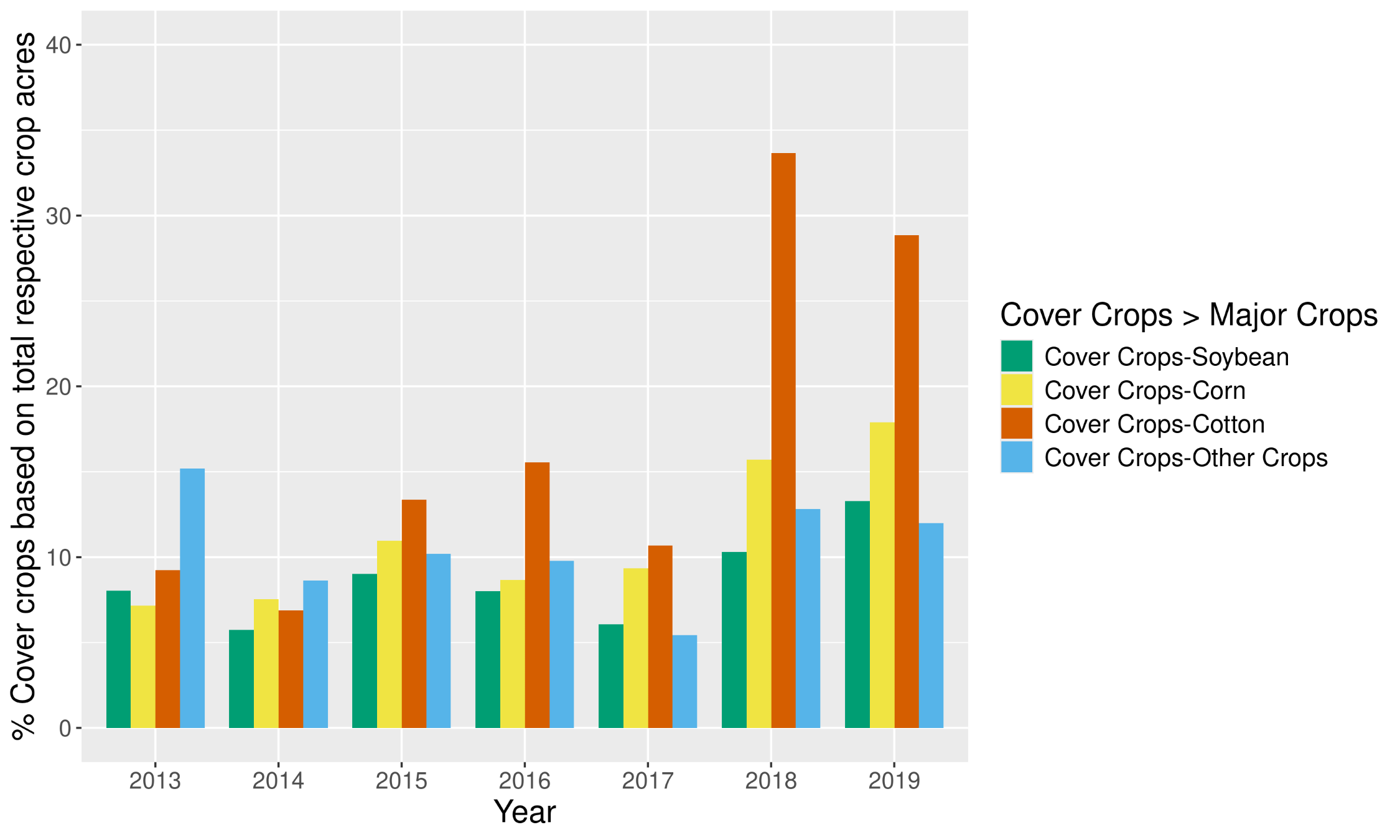

Supplement: S2 Fig — (TIF) [file pone.0331797.s002.tif]
