# Supplementary material for: Satellite Remote Sensing Reveals Voluntary Cover-Crop Adoption and Crop-Rotation Hotspots in the Mississippi Alluvial Plain
Source: PLoS One. 2025 Oct 21;20(10):e0331797. doi: 10.1371/journal.pone.0331797 (PMC12539724; doi:10.1371/journal.pone.0331797)
Supplement: S1 Table — (DOCX) [file pone.0331797.s003.docx]

| **Cropping Pattern** | Estimate | p-value | significant |
| --- | --- | --- | --- |
| Corn - None - Corn | NA | NA | NA |
| Corn - None - Cotton | NA | NA | NA |
| Corn - None - Soybean | -0.089 | 0.761 | No |
| Cotton - CC - Cotton | -0.5 | NA | NA |
| Cotton - None - Cotton | 0.288 | 0.027 | Yes |
| Cotton - None - Soybean | -0.287 | 0.033 | Yes |
| Double Crops - None - Corn | NA | NA | NA |
| Double Crops - None - Soybean | -0.71 | 0.128 | No |
| Minor Crops - CC - Minor Crops | -0.018 | 0.931 | No |
| Minor Crops - None - Minor Crops | 0.345 | 0.444 | No |
| Minor Crops - None - Rice | 0.295 | 0.532 | No |
| Minor Crops - None - Soybean | 0.284 | 0.026 | Yes |
| Rice - None - Minor Crops | 0.129 | 0.834 | No |
| Rice - None - Rice | -0.134 | 0.333 | No |
| Rice - None - Soybean | -0.15 | 0.672 | No |
| Sorghum - None - Soybean | NA | NA | NA |
| Soybean - CC - Minor Crops | NA | NA | NA |
| Soybean - CC - Soybean | 0.223 | 0.018 | Yes |
| Soybean - None - Corn | 0.266 | 0.29 | No |
| Soybean - None - Cotton | -0.011 | 0.945 | No |
| Soybean - None - Minor Crops | 0.407 | 0.418 | No |
| Soybean - None - Rice | -0.342 | 0.273 | No |
| Soybean - None - Sorghum | 2.86 | NA | NA |
| Soybean - None - Soybean | -0.007 | 0.994 | No |
